# A didactic approach to presenting verbal and visual information to children participating in research protocols: the comic book informed assent

**DOI:** 10.6061/clinics/2018/e207

**Published:** 2018-08-21

**Authors:** Thaís Massetti, Tânia Brusque Crocetta, Regiani Guarnieri, Talita Dias da Silva, Andrea Fernanda Leal, Mariana Callil Voos, Carlos Bandeira de Mello Monteiro

**Affiliations:** I Programa de Graduacao em Ciencias da Reabilitacao, Faculdade de Medicina, Universidade de Sao Paulo, Sao Paulo, SP, BR; II Laboratorio de Delineamento de Estudos e Escrita Cientifica, Faculdade de Medicina do ABC, Santo Andre, SP, BR; III EACH - Escola de Artes, Ciencias e Humanidades, Universidade de Sao Paulo, Sao Paulo, SP, BR

**Keywords:** Informed Assent, Informed Consent, Ethics Committee, Children, Parents

## Abstract

**OBJECTIVE:**

When children participate in research protocols, consent (by a parent or legal guardian) and assent (by the children) must be given. Understanding research protocols can be challenging for an adult and even more difficult for a child. The aim of this study was to describe the development of a comic book created to facilitate children's understanding of informed assent with clear and simple language.

**METHODS:**

Five researchers with scores above seven according to the Fehring criteria developed the comic book, avoiding the use of technical terminology. Twenty children between 7 and 12 years old, and enrolled in a larger study, responded using a Likert scale and questions about the clarity of texts and illustrations. The final version met National Health System Resolutions (Resoluções do Conselho Nacional da Saúde - CNS n° 196/1996 and 466/2012).

**RESULTS:**

The comic book assent presents a short story containing information about a real study: the invitation to participate, objectives, methods, instruments, procedures, risks, benefits, and the researchers' contact information. Most of the participants answered that they perceived the content of the text to be “Excellent” (40%) and “Very good” (40%), and the illustrations were perceived as “Excellent” (45%) and “Very good” (55%).

**CONCLUSION:**

The construction of a simple and clear model of informed assent is possible, and this model should be used in experiments with children.

## INTRODUCTION

Prior to the beginning of any study, all research protocols must be evaluated by an ethics committee. This requirement is designed to ensure integrity and that people are able to make informed decisions about their participation in research. Participants must know all procedures and risks involved 1 in research protocols. Thus, they must be provided with information, and then they can decide whether to participate in the study 2. To ensure that this right is respected, international documents and expressions, such as “informed consent”, were created and have been increasingly mandatory in research.

To obtain informed consent, researchers must clearly and objectively describe the study procedures to the participants and/or their guardians, and documentation must be signed by the researcher and the participant or their legal guardian. However, this process does not take into consideration the opinions of children, adolescents, or people who are legally incapable of understanding such procedures. Therefore, one of the main changes to the Resolution of the Brazilian National Health System “Conselho Nacional de Saúde CNS - Resolução n°” 466/2012 3, which replaced the Resolution “Resolução n°” 196/1996, was the inclusion of the term “free and informed assent”, according to which children should express their agreement 4 prior to participating in research protocols. Assent is the term used to convey a sense of agreement obtained from those who are not able to enter into a legal contract to participate in the study 5,6.

However, previous research has shown that disclosure for children is rarely adequate. The researcher may use difficult language, or there may be a general lack of informative materials specifically designed for children, 7-9 adolescents, or those who are legally incapable of assenting. Thus, these types of participants often fail to comprehend essential aspects of the research they are involved in 10. Several studies have corroborated this 11-14. For example, the clinical trial conducted by O'Lonergan and Forster-Harwood (2011) 15 showed that 76% of the children involved did not understand the risks associated with participation. In Chappuy et al. (2008) 16, 79% of the children did not understand the right to withdraw, and in Unguru et al. (2010) 8, 51% of the children did not know that their treatment was part of research. Tait et al. (2007) 17 state that 34.3% of the young children in their study reported that the standard form was difficult to read and had “too much” information, which seems to be the major obstacle to a child understanding an assent form.

All of these studies have shown that an improved understanding of study-specific research components (rather than research rights) may improve overall comprehension. Although the Research Ethics Committee (REC) requests that informed consent and informed assent should be easy to understand, it is questionable whether participants would be interested in reading such information even if it were presented in completely clear prose 18. One suggestion to encourage the reading and comprehension of assent forms is the use of pictures or cartoons. According to Tait et al. (2007) 17, 81% of young children chose a modified form with pictures that improved reading and comprehension. Comic books have been successfully used to educate stroke patients 19 and schoolchildren 10, and the deliberations regarding their importance and effectiveness justify the use of pictures or cartoons to enable children, adolescents, and the legally incapable to easily understand protocols, processes, risks, and the right to withdraw.

Informed assent must be developed in an empathic way and meet the ethical and legal requirements of research practice 4. A child who refuses to give his/her assent must be heard, particularly if the benefits of the treatment are uncertain 5,20, e.g., in progressive or severe diseases. Considering the need for terms of assent 21, the aim of this study is to develop and present informed assent in comic book form. The authors aimed to provide clear verbal and visual information to enable participants' understanding of research protocols prior to giving informed assent.

## METHOD

**Figure 1:**
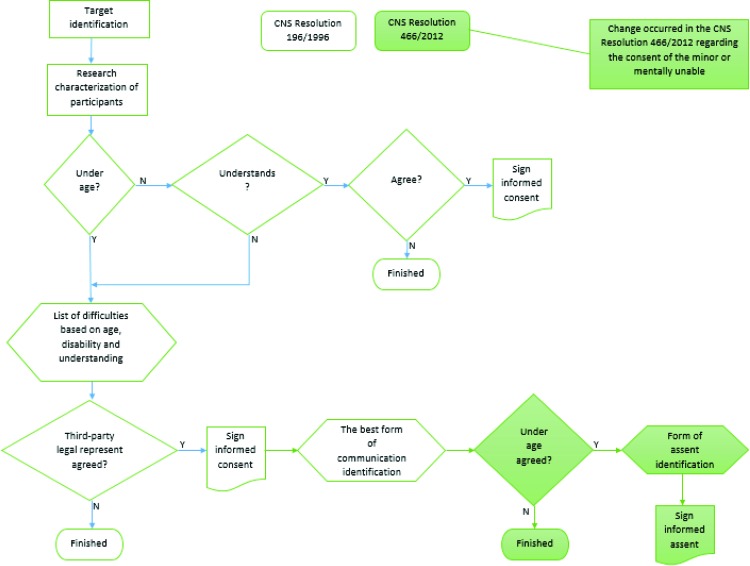
Flowchart for the identification of the target group for the research.

This study describes the development of an informed assent tool for children. The steps determined by the Brazilian National Health System “CNS - Resolução n° 466/2012” (Figure 1) were followed. With the goal of improving participants' understanding, a sequence was created for the construction of the assent form as a comic book.

**Effective term:** Based on the understanding of the children in the pilot group, the term was revised. The final version was approved by the Ethics Committee of the Faculty of Medicine of ABC – FMABC in March 2015, protocol number 39122214.6.0000.0082.

**Target identification:** The first step was to identify the target group for the research.

**Characterization of research participants:** The inclusion criteria of the study were boys and girls between 7 and 12 years old, who are considered children and adolescents according to the World Health Organization (WHO).

**List of difficulties based on age, disability, and understanding:** First, a form was developed for 7- to 12-year-old participants who could understand spoken and written Brazilian Portuguese (the assent form was translated into the English language for publication and international usability). The assent form was explained after parents or legal guardians had signed the informed consent form. Children sitting in regular chairs and in wheelchairs were included to make the protocol representative of typical children and children with motor disabilities.

**Best form of communication:** If children were unable to read the assent document independently, the documents could be read out loud for them, and verbal scripts could be used 6. The procedures were represented by comics with explanatory text, and these could be read aloud for each child.

**Identification of the accepted form of consent (signature, verbal, or positive nod):** Seven-year-old children or those who are legally incapable may be illiterate or in the process of developing literacy; thus, we accepted a signature or a positive nod to constitute consent.

**Proposed assembly:** The text was developed by five researchers, experts in clinical trials, who had scored an eight or higher based on the expert classification system of Fehring's criteria 22: i.e., master's degree (4 points), PhD (2 points), publications in peer reviewed journals (2 points), conducting research with the target public of the assent term (2 points) and one year of clinical experience (2 points). The drawings were presented in the form of comics, representing the main stages, instruments and procedures of a hypothetical project. The illustrations had the support of a professional designer who was responsible for the visual material contained in the form. The procedures were presented in the form of illustrations, showing each task being performed by a child. In some illustrations, the child was sitting on a wheelchair. The hypothetical project aimed to test the use of virtual reality games for motor learning and rehabilitation.

**Figure 2:**
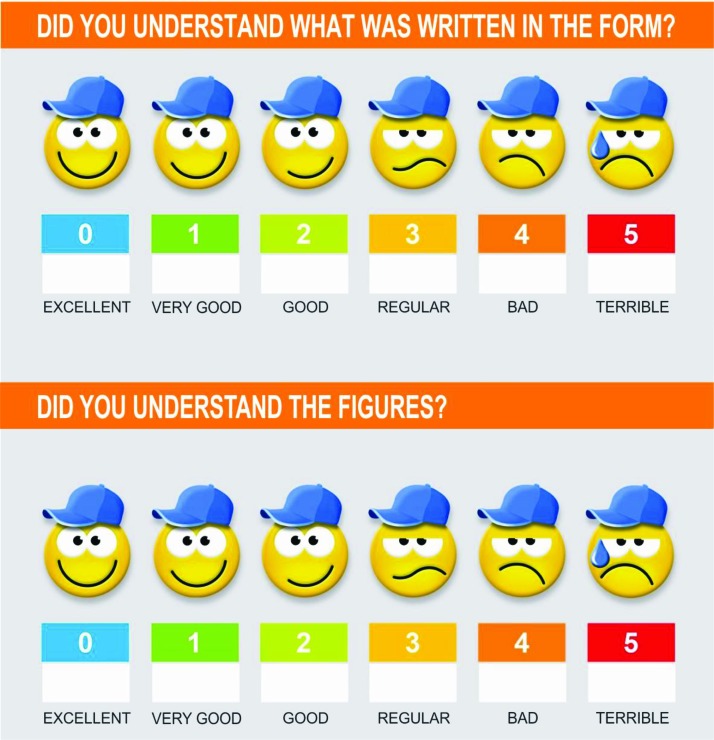
Likert scale questionnaire.

**Evaluating the pilot research:** A sample of 20 children between the ages of 7 and 12 years responded to the Likert-scale-based questionnaire represented by figure 2; the questionnaire focused on the understanding of verbal and visual information. These children are part of a larger study being conducted to evaluate performance in a computational task using the Check Limit game of the Team Bridge Games (described by Crocetta et al. 23) package with different movement acquisition interfaces. On the date of the present study, 27 children with cerebral palsy had been evaluated together with 30 children with typical development as control participants. The proposed informed assent was presented to all of the children. The first 20 children enrolled in the study were invited to respond to the Likert scale shortly after reading the consent form.

## RESULTS

The first result of this study is the proposed comic book assent form, which was created with clear and easy-to-understand text, using simple words and illustrations that represented the main points of the research. This comic book included the study objectives, procedures, risks, and the right to withdraw, following Brazilian Resolution “CNS - Resolução n° 466/2012”. The assent form is presented in Figures 3A-D. Figure 3A shows the first page of the form, which invites children to participate. Figures 3B and 3C show details about the instruments used, as well as some images of the games. The emphasis is on the presentation of the research materials and procedures. Figure 3D shows the last page of the form, which presents the researchers' contact information and the directions for the necessary signatures. The texts are short, but they present the nature of the research, as well as its objectives, methods (procedures), expected benefits, and options for receiving assistance to address the possible risks or inconveniences that participants may experience. The figures support the understanding of this information.

After receiving the information, 20 healthy children evaluated the assent form with two Likert scales (Figure 2). Children gave scores of 0 (n=8; 40%), 1 (n=8; 40%), and 2 (n=4; 20%) on the first Likert scale (“Did you understand what was written in the form?”), and they gave scores of 0 (n=9; 45%) and 1 (n=11; 55%) on the second Likert scale (“Did you understand the figures?”).

## DISCUSSION

Although consent is obtained from parents or legal guardians, obtaining informed assent from children participating in research is also important 6. New approaches should be developed to facilitate children's comprehension of a study's protocols or intervention procedures. According to Eisenberg and Anderson 18, legal documents do not need to be complicated; they just need to satisfy the law. Documents that are clear and understandable can still obey the law. This idea is corroborated by the fact that other comic book initiatives around the world 11-14 have previously been used successfully to educate patients (both children and adults) 10,19. The present study further supports those findings and demonstrates that all 20 healthy children rated their understanding of the comic book's text as either perfect, very good, or good, and their understanding of the illustrations was rated as perfect and very good. Thus, the authors of this study conclude that the use of comic books for consent and assent should be further explored: the comic book approach is easy to understand and increases participants' levels of engagement; people were more interested in listening and reading, and this interest increases their comprehension of the study's purpose, procedures, and risks.

Clinical practitioners Furuno and Sasajima 19 believe that a gap still exists between physicians and patients with regard to understanding clinical conditions. Thus, they created comics to explain diseases to patients (in this case, subarachnoid hemorrhage and intracerebral hemorrhage). Furuno and Sasajima 19 noted that the advantage of comic books is that participants can read them over and over after listening to the doctor's explanation: “We thought that visual and narrative illustration in comics would be more useful to patients and their families than an extensive explanation conducted by a physician”. For example, they state that comics could function as a new communication tool for informed consent between doctor and patient.

This study demonstrates how essential verbal and visual information for an assent form can be presented as a comic book to facilitate reading and understanding by 7 to 12-year-old children. According to Piaget 24, seven-year-old children are at the beginning of the concrete operational stage, which lasts until they are 11 or 12 years old. During this period, the child can contrast and compare objects and events. Therefore, children begin to think more logically, but their thinking can also be very rigid. However, they tend to struggle with abstract concepts, and illustrations can thus help with their understanding.

The ability to understand the consequences of their actions and those of others is a process that usually starts at the age of six and matures in late adolescence 25. Thus, children have the right to make choices about diagnostic and therapeutic procedures, even if the consent of their parents or legal guardians is obtained prior to any study 20.

It is the belief of the authors of this study that presenting informed assent in comic book form improved comprehension of the research protocol. Given the difficulty that children may have in understanding informed assent, information must be provided in a manner appropriate for the participant's age and level of knowledge. Related risks and benefits must be addressed. Given that children may be embarrassed to ask questions, it was supposed that illustrations and short pieces of text could clarify information about the experiment 26.

According to CAPPesq (Ethics Committee for Project Analysis of the University of São Paulo), the lack of clarity and objectivity in informed consent forms are the main reasons that projects are rejected by ethics committees (this problem accounts for approximately 5% of non-approvals for analyzed projects) 27. Furthermore, REC reviews the terms of informed consent and informed assent forms 27 to ensure that the ethical and methodological aspects are appropriate 28. Few studies have investigated the level of understanding required by informed consent and assent forms, 12-14 and the validity of these forms is questionable if the participants do not understand what they have read 26.

Ford, Sankey, and Crisp 6 described the processes used to include children in developing both a research information sheet and an assent form for research into children's understandings of their hospital experiences. The authors developed documents that were more understandable for their intended audience. REC also noted the need to have a specific term for children's assent, according to which the children could receive direct information about what was being done. Some strategies have been used to improve the quality of the process of obtaining and clarifying the informed consent and assent documents. Properly informed individuals are able to freely exercise their choice of whether to participate in research, and increased clarity improves the overall quality of the consent obtained 29.

Informed consent is a very complex process and is not simply a form that must be signed, especially considering the large amount of information that must be provided 30. This process is even more challenging when the participant is a child. Some studies have noted the terms and items required to build the assent document 4,21, but researchers need to be concerned about the clarity and accessibility of the language used. The present study proposed an assent form in the form of a comic book that contained both verbal and visual information; such an approach can be a way to comply with Resolution No. 466/2012 3.

Based on the discussion above, the authors of this study believe that considering the need for children to understand the procedures that involve their participation in research, and especially considering their rights, assent in comic book form is an excellent tool that can be easily used and reproduced around the world.

### Limitations and future studies

The current study has some limitations that are important to note. The first limitation is the number of children analyzed (only 20 children), the second limitation is the use of only two Likert scale questions; more questions could have enabled a stronger interpretation. Thus, it is recommended that future studies analyze a larger number of participants, a wider span of age ranges, and participants with a range of different disabilities. A third limitation is that no instrument was used to evaluate the impact of the proposed assent form on the children's adherence.

Perhaps the most important limitation of this study was the text developed by the researchers, which was created without the direct involvement of the children in the study. The children enrolled were only asked about their understanding and not about possible improvements to the composition of the text.

Informed assent must contain information that facilitates the understanding of a research participant, especially when the research involves children. This article presented the development of an assent form; the invitation to participate, as well as the objectives, methods, instruments, procedures, risks, benefits, and contact information, were all presented in comic book form. This is a coherent and clear model of informed assent that can be used by children participating in research projects.

## AUTHOR CONTRIBUTIONS

Massetti T structured the manuscript and directed the work. Massetti T, Crocetta TB and Guarnieri R performed the data collection and organized the data. Silva TD structured the method and data analysis. Leal AF and Massetti T structured the discussion and conclusion. Monteiro CB performed the data analysis and set up the work of the results. Voos MC and Crocetta TB adapted the work to the English language. Silva TD and Guarnieri R helped in the construction of the discussion. Monteiro CB revised and organized the manuscript.

## Figures and Tables

**Figure 3a:**
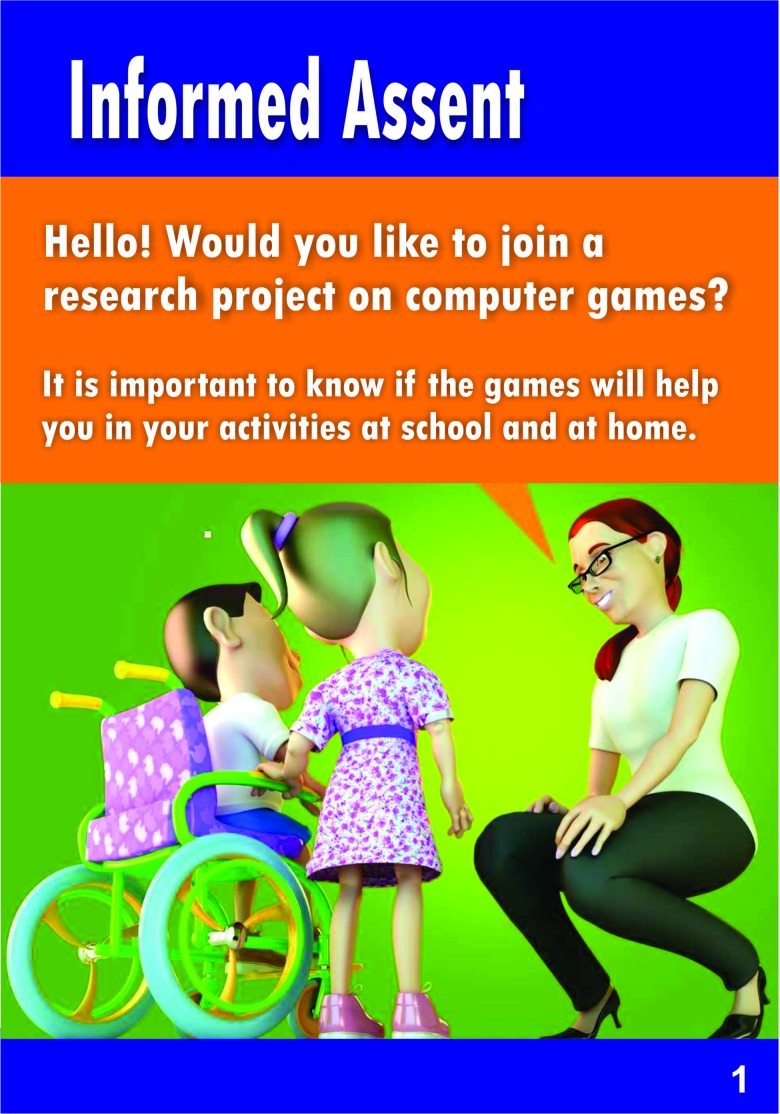
Model of assent - Part A.

**Figure 3b:**
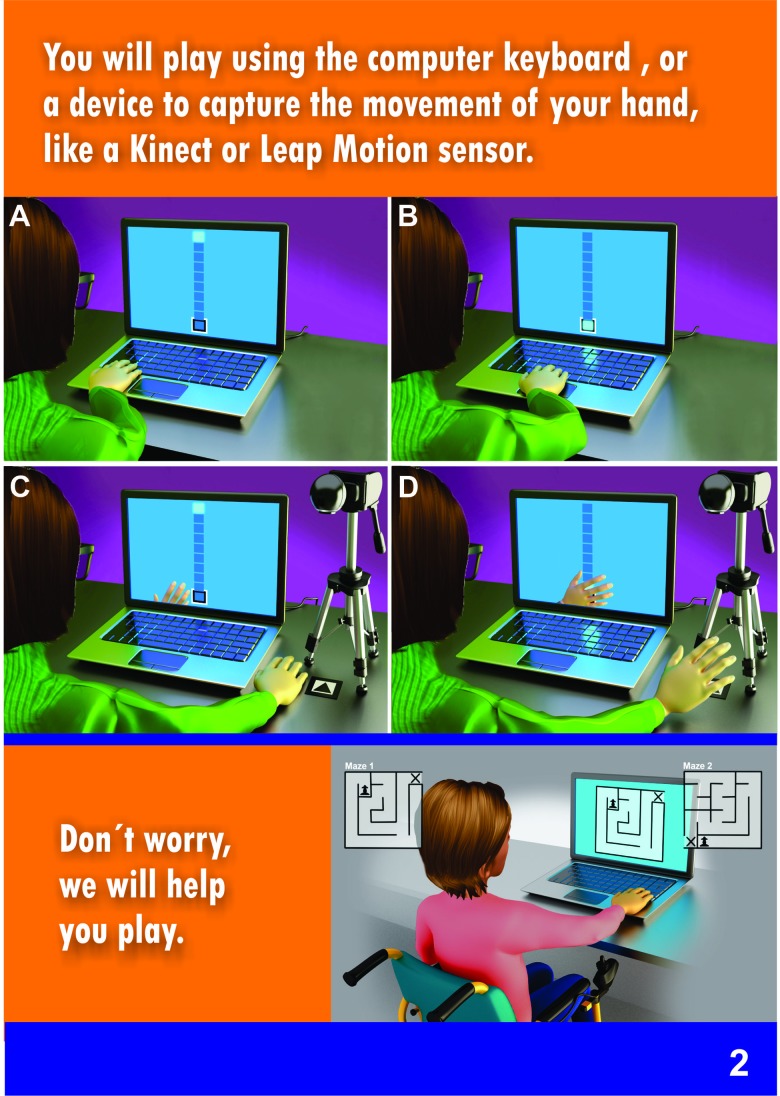
Model of assent - Part B.

**Figure 3c:**
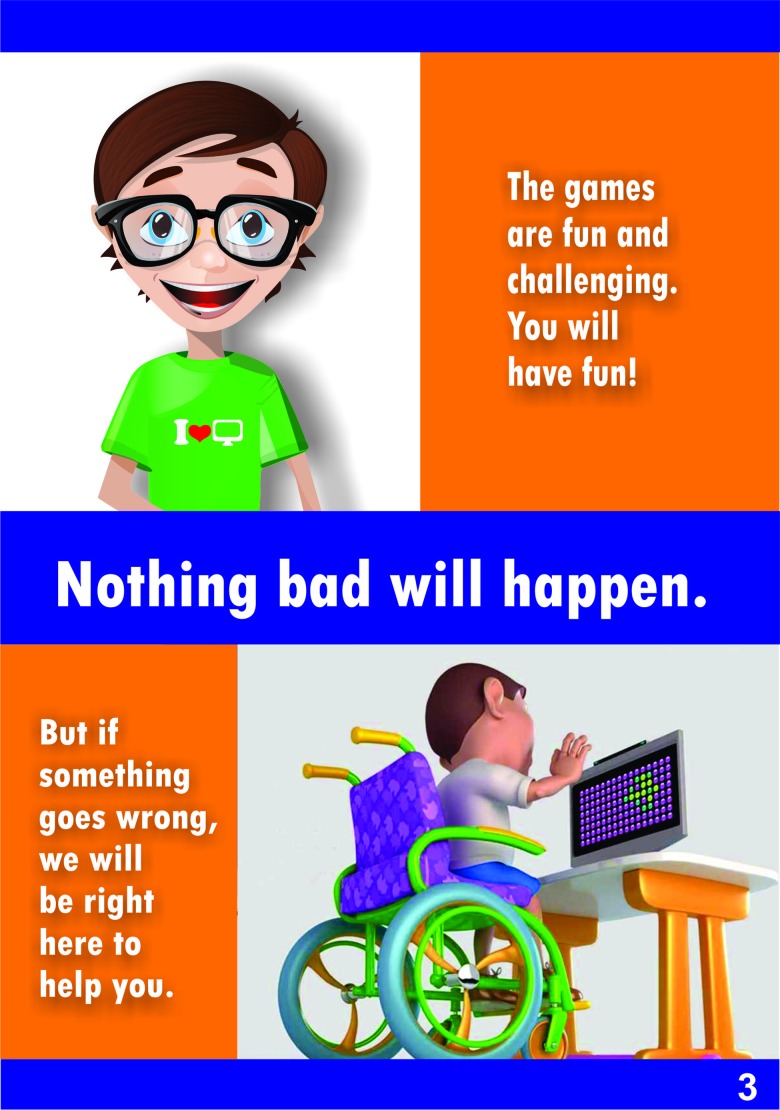
Model of assent - Part C.

**Figure 3d:**
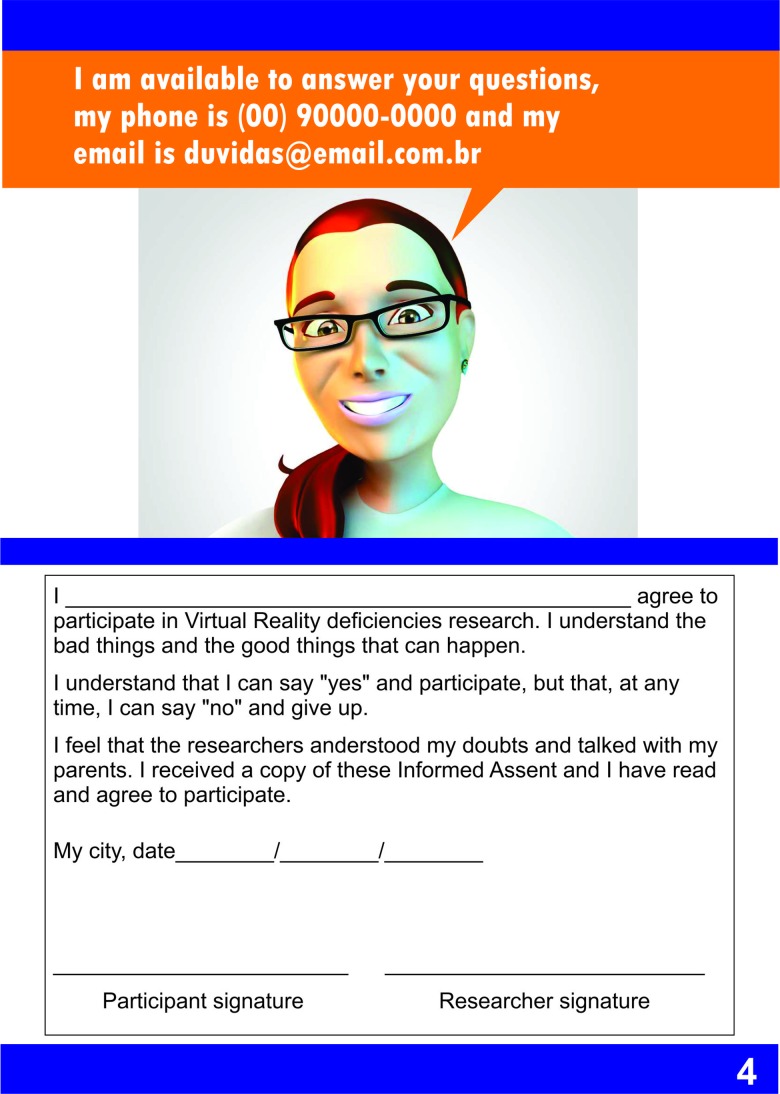
Model of assent - Part D.
